# 253 Implementation and dissemination of physical activity-related health competence in nursing education: First results of a hybrid type 2 effectiveness-implementation study

**DOI:** 10.1093/eurpub/ckae114.277

**Published:** 2024-09-26

**Authors:** Eva Grüne, Johanna Popp, Johannes Carl, Verena Hartung, Klaus Pfeifer

**Affiliations:** Friedrich-Alexander-Universität Erlangen-Nürnberg, Erlangen, Germany; Friedrich-Alexander-Universität Erlangen-Nürnberg, Erlangen, Germany; Friedrich-Alexander-Universität Erlangen-Nürnberg, Erlangen, Germany; Deakin University, Geelong, Australia; Friedrich-Alexander-Universität Erlangen-Nürnberg, Erlangen, Germany; Friedrich-Alexander-Universität Erlangen-Nürnberg, Erlangen, Germany

**Keywords:** Cluster-randomized controlled trial, Vocational education and training, Schools, Co-Creation, Intervention Mapping

## Abstract

**Background:**

Acknowledging the health-enhancing effects of physical activity (PA), the nursing education system in Bavaria, Germany, has recently integrated the promotion of physical activity-related health competence (PAHCO) into the curriculum. However, it cannot be assumed that PAHCO has sufficiently permeated the educational practices and routines of nursing schools. Therefore, the aim of the TakeCare! project is to evaluate the effectiveness and implementation of different intervention approaches to address PAHCO in nursing education.

**Methods:**

Within a hybrid type 2 effectiveness-implementation study, we conduct a cluster-randomized controlled trial with 16 nursing schools allocated to four parallel study arms to determine the most appropriate approach to strengthen PAHCO. While intervention group 1 applies a bottom-up approach involving various stakeholders in the participatory development of PA-promoting interventions, intervention groups 2 and 3 follow a top-down approach in which the intervention is developed using intervention mapping and delivered by external PA professionals or nursing teachers respectively. The control group follows regular curriculum.

To investigate intervention implementation, questionnaires and protocol data are analysed using descriptive distributions and content analyses. To examine the short-term effectiveness, questionnaire-based data on nursing students’ PA and PAHCO collected before (T0/T1) and after intervention implementation (T2) are analysed via linear models.

**Results:**

The implementation evaluation shows that interventions were implemented in each school across the different intervention groups. In intervention group 1, each school developed different intervention components (4≤*n*≤8), some of which were initiated (1≤*n*≤3). In the intervention groups 2 and 3, the intervention units developed (*N*=12) were largely implemented as planned. While six external PA professionals carried out all intervention units in the schools of intervention group 2, nine nursing teachers carried out the majority of intervention units (*M*=10.8) in the schools of intervention group 3. The effectiveness evaluation has not yet been finalised.

**Discussion:**

The TakeCare! study provides valuable insights into the comparison of different intervention approaches to promote PAHCO and PA in terms of their effectiveness and implementation and enables evidence-based decision when choosing a suitable intervention approach. To spread PAHCO in all 185 Bavarian nursing schools, a systematic dissemination strategy must be developed based on the findings.

